# Genomic organization of duplicated major histocompatibility complex class I regions in Atlantic salmon (*Salmo salar*)

**DOI:** 10.1186/1471-2164-8-251

**Published:** 2007-07-25

**Authors:** Morten F Lukacs, Håvard Harstad, Unni Grimholt, Marianne Beetz-Sargent, Glenn A Cooper, Linda Reid, Hege G Bakke, Ruth B Phillips, Kristina M Miller, William S Davidson, Ben F Koop

**Affiliations:** 1 Department of Basic Science and Aquatic Medicine, Norwegian School of Veterinary Science, Oslo, Norway; 2 Department of Biology, University of Victoria, Victoria BC V8W 2Y2, Canada; 3 Biological Sciences, Washington State University Vancouver, Vancouver, Washington, USA; 4 Molecular Genetics, Pacific Biological Station, Fisheries and Oceans Canada, Nanaimo, BC, Canada; 5 Department of Molecular Biology & Biochemistry, Simon Fraser University, Burnaby, BC, Canada

## Abstract

**Background:**

We have previously identified associations between major histocompatibility complex (MHC) class I and resistance towards bacterial and viral pathogens in Atlantic salmon. To evaluate if only MHC or also closely linked genes contributed to the observed resistance we ventured into sequencing of the duplicated MHC class I regions of Atlantic salmon.

**Results:**

Nine BACs covering more than 500 kb of the two duplicated MHC class I regions of Atlantic salmon were sequenced and the gene organizations characterized. Both regions contained the proteasome components PSMB8, PSMB9, PSMB9-like and PSMB10 in addition to the transporter for antigen processing TAP2, as well as genes for KIFC1, ZBTB22, DAXX, TAPBP, BRD2, COL11A2, RXRB and SLC39A7. The IA region contained the recently reported MHC class I *Sasa-ULA *locus residing approximately 50 kb upstream of the major *Sasa-UBA *locus. The duplicated class IB region contained an MHC class I locus resembling the rainbow trout *UCA *locus, but although transcribed it was a pseudogene. No other MHC class I-like genes were detected in the two duplicated regions. Two allelic BACs spanning the *UBA *locus had 99.2% identity over 125 kb, while the IA region showed 82.5% identity over 136 kb to the IB region. The Atlantic salmon IB region had an insert of 220 kb in comparison to the IA region containing three chitin synthase genes.

**Conclusion:**

We have characterized the gene organization of more than 500 kb of the two duplicated MHC class I regions in Atlantic salmon. Although Atlantic salmon and rainbow trout are closely related, the gene organization of their IB region has undergone extensive gene rearrangements. The Atlantic salmon has only one class I *UCA *pseudogene in the IB region while trout contains the four MHC *UCA*, *UDA*, *UEA *and *UFA *class I loci. The large differences in gene content and most likely function of the salmon and trout class IB region clearly argues that sequencing of salmon will not necessarily provide information relevant for trout and vice versa.

## Background

Major histocompatibility complex (MHC) class I and class II molecules are vital parts of the cellular immune system presenting self and/or foreign peptides to CD8 positive and CD4 positive T cells. Both classes of genes reside in a 4 Mb gene dense region on human chromosome 6 shared with many other immune genes [1].

Atlantic salmon and rainbow trout genomes encode one major MHC class I locus designated *UBA *in addition to the major MHC class II alpha and beta genes designated *DAA *and *DAB *respectively [2-4]. For *UBA*, the main polymorphism resides in the alpha 1 and alpha 2 domains with up to 60% sequence divergence between these antigen binding domains. Added variability for *UBA *is produced by shuffling of exon 2 onto different exon 3 and downstream regions through recombination occurring in intron 2 [4]. Additional class I loci and lineages have been described in both Atlantic salmon as well as in rainbow trout. The majority of reported salmonid MHC class I molecules are classified into a U-lineage consisting of both *UBA *as well as non-classical MHC molecules [5,6]. Two other described MHC class I-like lineages are ZE described by Miller *et al*. [5] and L described by Dijkstra *et al*. [7].

In all teleosts studied so far including salmonids the MHC class I and class II regions are unlinked [3,8]. Sequence data on the MHC class I region is available from zebrafish [9], fugu [10], medaka [11,12] and rainbow trout [6]. A general feature of these four MHC class I regions is a core region containing genes for the proteasome components (PSMBs) and the transporter for antigen processing (TAP2) being flanked by various numbers of MHC class I loci in addition to many other genes also residing in the human MHC region located on chromosome 6. Data from medaka and zebrafish indicate that other fish orthologs of the mammalian MHC-encoded genes are dispersed on several different chromosomes [13-16], similar to the paralogue MHC regions described on human chromosomes 1, 9 and 19 [17]. Salmonids are seen as partially tetraploid with a unique whole genome duplication occurring between 25 and 125 million years ago (mya) with remnants of tetraploidy visible also today [18-20]. Shiina *et al*. [6] sequenced two duplicated core MHC regions of rainbow trout. Based on sequence divergence they estimated the duplication event to have taken place approx. 60 mya, in agreement with the salmonid whole genome duplication theory. The classical or IA region contained the major expressed classical MHC class I *UBA *locus while the duplicated region denoted IB contained the four *Onmy*-*UCA*, -*UDA*, -*UEA *and -*UFA *class I loci. Based on expression and polymorphism data, *Onmy-UCA*, -*UDA *and -*UEA *were defined as non-classical loci and -*UFA *as a pseudogene due to an incapacitating mutation in exon 3 [6].

Data is rapidly emerging on associations between MHC and resistance to salmonid pathogens. In Atlantic salmon, *UBA *genotypes have been found to provide resistance towards *Aeromonas salmonicida *and Infectious Salmon Anaemia Virus [21,22]. Class IB, but not class IA was found associated with susceptibility towards infectious hematopoietic necrosis virus (IHNV) in Atlantic salmon and towards infectious pancreatic necrosis virus (IPNV) in rainbow trout [23,24].

Both trout and salmon are main aquaculture species and understanding their immune systems will improve our understanding of how these regions influence disease resistance and thus improve our breeding schemes for the trait. Atlantic salmon and rainbow trout are estimated to have split approx. 20 mya [25]. As Atlantic salmon is a major aquaculture species and displays some differences in response to pathogens when compared to rainbow trout [26], we ventured into sequencing of the two duplicated MHC class I regions of Atlantic salmon. Here we describe the gene organization of these two MHC class I regions comprising approx. 500 kb each and compare our results to data from other teleosts.

## Results and discussion

The aim of this study was to characterize the gene organization and identify new genes potentially contributing to disease resistance in the two MHC class I regions of Atlantic salmon.

### Characterization and sequencing of BAC clones

*Sasa-UBA *and TAP2 probes hybridized to 74 BAC clones, where 18 clones were positive for both probes. The 74 BAC clones were ordered into three contigs using restriction fragment analysis together with GRASP *Hind*III fingerprint information [27].

The two contigs that were positive for *UBA*, TAP2, PSMB9 and PSMB8 by southern hybridization, were tested for presence of a polymorphic dinucleotide repeat located in the 3'UTR of the *UBA *locus [3]. Only BAC clones from one of the two contigs gave PCR-products, thus this contig was defined as the IA region, and the other contig remained a candidate for the duplicated IB region. The BAC clones in the third contig hybridized to the *UBA *probe as well as a mixed *UBA *exon 2 probe. These clones also tested positive for a U-lineage *ULA *locus that has previously been found closely linked to *UBA *[5].

Three BACs were sequenced from the IA region. The BAC clones 92I04 and 714P22 indicated allelic variants based on variation in the *UBA *3'UTR marker (data not shown) with 523M19 as a continuation of 714P22. From the duplicated IB region we chose 8I14, 424M17, 15L20 and 189M18 for sequencing. 30C23 was chosen as a candidate from the third contig and was extended 5 kb with the sequence of 868O01. The selected BAC clones were subcloned, sequenced, and assembled into continuous sequences. The Atlantic salmon IA region consisted of the BAC clones 30C23, 868O01, 92I04, 714P22 and 523M19 covering 502869 bp, while the IB region consisted of 8I14, 424M17, 15L20 and 189M18 totaling 522617 bp.

### Gene organization of the Atlantic salmon MHC class I regions

We have adopted the nomenclature described by Shiina *et al*. [6] with IA covering the *UBA *locus region and IB for the duplicated region. Thus the genes identified in the regions will be named accordingly; the IA proteasome subunits are given an extension of a (PSMB9a) and the IB genes have an extension of b (PSMB9b). The previous symbol ABCB3 has been withdrawn for the transporter for antigen processing 2, so we have used the current symbol TAP2 [28].

**Figure 1:**
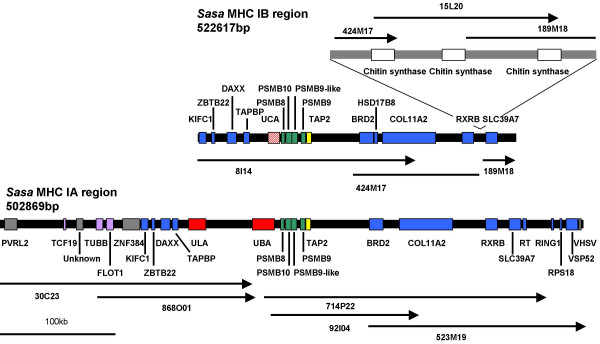
**Gene organization of Atlantic salmon MHC IA and IB regions**. Upper panel is the IB region represented by the BACs 8I14, 424M17, 15L20 and 189M18. Lower panel is the IA region represented by the BACs 30C23, 868O01, 92I04, 714P22 and 523M19. Locus designation is based on sequence identity to matching ESTs and human nomenclature is used. The regions are drawn to scale.

**Figure 2:**
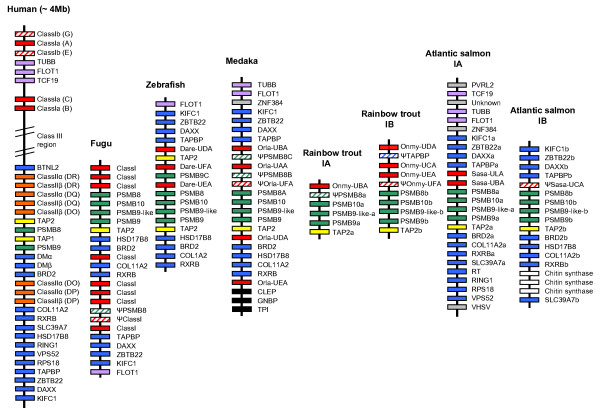
**Comparison of the human, Atlantic salmon, rainbow trout, medaka, zebrafish and fugu MHC class I**. Color code: red is MHC class I genes, orange is MHC class II genes, yellow is TAP genes, green is proteasome genes, blue is human extended MHC class II region genes, purple is human class I region genes, grey is non-human class I region genes, black is genes unique to the medaka HN1 strain [12]. Pseudogenes are striped. Human class III region genes are not shown. References are: zebrafish [9,16], fugu [10], medaka [11,12], rainbow trout [6] and human [1].

The gene organization of the IA and IB MHC regions is shown in Fig. 1. A core region was identified in both regions which included MHC class I genes, together with the proteasome subunits genes *PSMB8 *(*LMP7*), *PSMB10 *(*MECL-1*), *PSMB9-like *(*LMP2-δ*), *PSMB9 *(*LMP2*) and *TAP2*. The gene order and orientation of the Atlantic salmon PSMBs and TAP2 was very similar to that found in rainbow trout and other teleost (Fig. 2). For the IA region, the main difference between Atlantic salmon and rainbow trout is that the rainbow trout PSMB8a gene is a pseudogene.

The IA region contained the major MHC class I *Sasa-UBA *locus and the recently reported *Sasa-ULA *locus residing approximately 50 kb upstream. The duplicated class IB region contained an MHC class I locus resembling the rainbow trout *UCA *locus, but although transcribed it was a pseudogene. No other MHC class I-like genes were detected in the two duplicated regions.

Outside the core region we found 12 Atlantic salmon orthologs of genes residing in the extended human MHC class II region. Alternative nomenclature for these genes is described in Table 1. The following genes were found in both the IA and IB regions; KIFC1, ZBTB22, DAXX, TAPBP, BRD2, COL11A2, RXRB and SLC39A7. Three orthologs found in the IA region only were RING1, RPS18 and VPS52. For other teleosts, the gene organization of the extended MHC class I region is partly known for zebrafish [9,16], fugu [10,15] and medaka [11,12]. The TAPBP, DAXX, ZBTB22 and KIFC1 genes are conserved in the same order in both fish and human (Fig. 2). As described in medaka we also found a gene for ZNF384 in the IA region, which is located on Chromosome 12 in human.

**Table 1:** EST match to genes in the Atlantic salmon MHC IA and IB regions

GENE	Abbreviation	Alias	EST/cDNA match IA	EST/cDNA match IB
Poliovirus receptor like2	PVRL2		CA342790	n.i.
Transcription factor 19	TCF19	SC1	?	n.i.
UNKNOWN	?		DW569240	n.i.
Tubulin	TUBB	OK/SW-cl.56	DW589685	n.i.
Flotillin	FLOT1		DY703577	n.i.
Zinc finger protein 384	ZNF384	CIZ	CB506768	n.i.
Kinesin family member C1	KIFC1	KNSL2/HSET	DW541824	BX313539
Zinc finger and BTB domain	ZBTB22	ZNF297/BING1	BX911712	BX911712
Death-associated protein 6	DAXX		DY736372	GRASP cluster 76574
Tapasin	TAPBP		DY735080	DW580568
MHC class I	ULA		DY699730	n.i.
MHC class I	UCA		n.i.	DW563256
MHC class I	UBA		UBA*0201/*0601	n.i.
Proteasome subunit, beta type, 8	PSMB8	LMP7	DY733578	AF184938
Proteasome subunit, beta type, 10	PSMB10	MECL1	DY740375	DY734168
Proteasome subunit, beta type, 9-like	PSMB9-like	LMP2-delta	DW574810	CN442539
Proteasome subunit, beta type, 9	PSMB9	LMP2	AF184935	AF184934
Transporter 2, ATP-binding cassette, sub-family B	TAP2	ABCB3	DW540744	Z83328
Bromodomain containing 2	BRD2	RING3	CA349460	CA349460
Hydroxysteroid (17-beta) dehydrogenase 8	HSD17B8	KE6, FABGL	n.i.	CK880913
Collagen, type XI, alpha 2	COL11A2		?	?
Retinoid × receptor, beta	RXRB		CK879311	CK357003
Chitin synthase			n.i.	DW550858
Solute carrier family 39 (zinc transporter), member 7	SLC39A7	KE4, FABGL	DW552689	DW563433
Reverse transcriptase	RT		DW561676	n.i.
Ring finger protein 1	RING1	RNF1	DY732022	n.i.
Ribosomal protein S18	RPS18	KE3	DY729361	n.i.
Vacuolar protein sorting 52	VSP52	SAC2	DY740244	n.i.
VHSV induced gene	VHSV		DW547400	n.i.

Mostly one match is listed per gene. Current abbreviation vs. older nomenclature is listed for each gene. All EST/cDNA sequences are from Genbank (? denotes unidentified EST, n.i. denotes not identified).

HSD17B8, which resides in between SLC39A7 and RING1 in the extended human class II region, was found in the IB region only and showed more than 81% identity towards counterparts in tilapia [Genbank:AAV74184], zebrafish [Genbank:CAK04961] and medaka [Genbank:BAB83840]. HSD17B8 has thus been deleted from the Atlantic salmon IA region as it is also present in other fish MHC class I regions (Fig. 2).

Three orthologs of genes located in the human class I region were identified in the IA region; TCF19, TUBB and FLOT1. Atlantic salmon tubulin is highly conserved and showed more than 94% identity towards mammalian counterparts. Another highly conserved gene is RPS18, which showed 98% identity towards mammalian sequences.

A gene that was predicted by DIGIT in the IA region had one EST match [Genbank:DW569240], but no homology to annotated proteins and is thus denoted unknown in Fig. 1. However, some sequence identity was found towards a protein in zebrafish located on chromosome 19 [Genbank:XP_001344849] as well as to a tetraodon nigroviridis protein [Genbank:CAF97811], which could indicate a molecule unique to teleosts.

**Figure 3:**
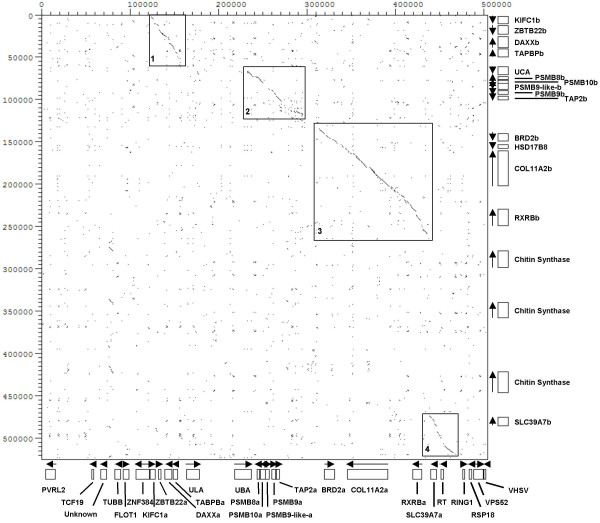
**Dot-plot analysis of the Atlantic salmon MHC IA and IB regions**. The IA region is a collection of the BAC clones 30C23, 868O01, 714P22 and 523M19. The IB region is a summary of the BAC sequences 8I14, 424M17, 15L20 and 189M18. Position of genes is shown on the right (IB) and below (IA), and direction of transcription is marked with arrows. Genes found in both regions have an a or b extension.

In addition to the genes described above we identified genes for PVRL2, RT, VSHV-induced gene and a novel gene similar to a non-vertebrate chitin synthase protein that are not MHC linked in humans. The human PVRL2 is located on chromosome 19 (19q13.2-q13.4). A homologue of this gene is also found on zebrafish chromosome 19 [Genbank:XP_689425]. A 220 kb insertion was found in the IB region in between the RXRB and SLC39A7 genes containing three copies of a chitin synthase gene approx. 45 kb apart (Figs. 1 and 3). Chitin synthase is involved in the synthesis of chitin, which is a main structural component of the fungal cell wall. A similar protein has also been identified in zebrafish [Genbank:CAK04859]. No chitin synthase genes were present in the IA region nor are chitin synthase genes found in any other teleost MHC regions suggestive of a single insertion of this gene in the IB region with two subsequent duplications (Fig. 2).

Most genes in both regions are supported by matching cDNAs apart from TCF19 and COL11A2 where no match has been found so far (Table 1). Other open reading frames were also identified, but were associated with transposon related repetitive elements.

### Comparison of the IA and IB regions

The two allelic BACs 92I04 and 714P22 had an overall sequences identity of 99.2% over 124574 bp, with similar exon intron organization for all genes. The major differences between the two allelic regions resided in the *UBA *α2 and α3 exons and in differences in number of repeats (data not shown). Dotplot analysis of 714P22 or 92I04 against themselves showed no extended regions of local similarity, with the exception of the *TAP2 *region which showed similarity due to a duplicated TAP2 exon 11 (data not shown).

A dot plot analysis of more than 500 kb of the IA and IB regions showed four regions with high sequence similarity consisting of subregion one containing genes for KIFC1 to TAPBP, subregion two ranging from PSMB8 to TAP2, subregion three covering BRD2 to RXRB and subregion four containing SLC39A7 (Fig. 3). The conserved regions in IA and IB have 82.5% identity over 136104 bp. In total, repeats constituted approximately 24% of the sequence in both regions, and 17% of the repeats were fish-specific DNA elements.

### MHC Class I genes

#### *Sasa-UBA*

**Figure 4:**
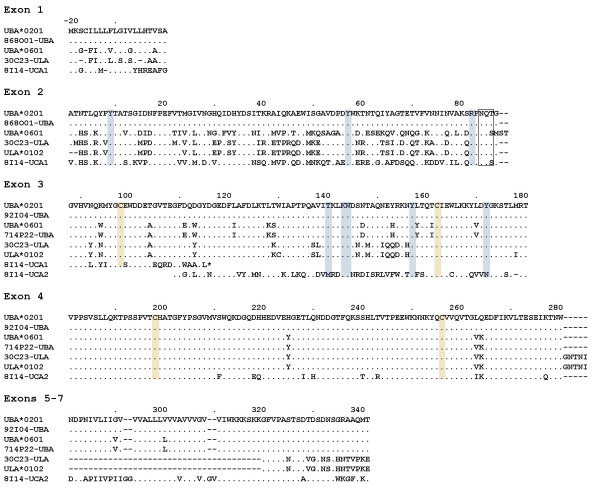
**Alignment of Atlantic salmon *UBA, ULA and UCA *alleles**. Comparison of Atlantic salmon *UBA*, *ULA *and the two reading frames of *UCA *sequences found in the BACs. Also shown is UBA*0201 [Genbank:AAN75117] with exon 1 sequence from [Genbank:DY698957], UBA*0601 [Genbank:AAN75107] with exon 1 sequence from [Genbank: DW579225] and ULA*0102 [Genbank:AAZ73115]. Residues critical for binding of peptide anchors are in blue, and disulfide-bridge cysteins are in orange while the glycosylation site is boxed. Dots indicate identity while dashes indicate missing residues.

The promoter, leader and α1 exons of *Sasa-UBA *were identified in 30C23/868O01, while the remaining exons of *Sasa-UBA *were found in 92I04 and 714P22. The leader and α1 exons found in 30C23/868O01 were identical to the PCR amplified UBA*0201 allele [Genbank:AF504023] as well as to the leader and α1 exons of another salmon full-length cDNA [Genbank:DY698957]. Together with the α2 and α3 exons of 92I04 they collectively provide a bona fida UBA*0201 allele. The *UBA *α2 exon and downstream sequences of the two allelic BACs 92I04 and 714P22 have complete sequence identity to the Sasa-UBA*0201/*0301 and Sasa-UBA*0601 alleles respectively. UBA*0201 and UBA*0301 are prime examples of the recombination shown to occur within intron 2 of salmonid *UBA *alleles [4] showing complete sequence identity in the α2 and downstream regions, but highly divergent α1 exons. The predicted amino acid sequences of *UBA*, *ULA *and the two open reading frames of *UCAψ *were aligned for comparison of the MHC class I genes encoded in the two regions (Fig. 4).

**Figure 5:**
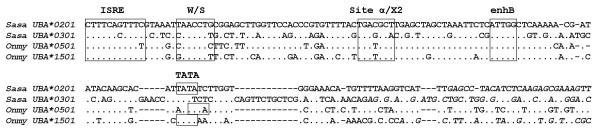
**Comparison of UBA promoter elements**. Comparison of the SasaUBA*0201 promoter region with other reported UBA promoters in Atlantic salmon and rainbow trout; SasaUBA*0301 [Genbank:DQ243891], OnmyUBA*0501 [Genbank:AB162342] and OnmyUBA*1501 [Genbank:AF441856]. Promoter elements are boxed [31,32] and the genomic sequences with complete identity to 5'UTRs of *UBA *cDNA alleles are indicated in italics. Dots indicate identity while dashes indicate gaps.

Analysis of the promoter sequence of UBA*0201 in 30C23/868O01 showed high similarity to a rainbow trout UBA*1501 promoter [29], both containing similar regulatory elements typical for MHC class I promoters such as an interferon stimulated response element (ISRE), W/S-box and enhancer B (enhB) (Fig. 5). The UBA*0201 promoter contains a potential site α element according to the core sequence (TGACGC) [30] while a sequence more resembling an X2-box has been found in rainbow trout (TGAGGCA). Both the site α and the homologous X2-box found in mammalian MHC class I and MHC class II promoters respectively, are involved in regulation of transcription and bind ATF/CREB family transcription factors [31]. A potential TATA-box was also identified in the promoter sequence of UBA*0201 [32]. A salmon UBA*0301 promoter published by Jorgensen *et al*. [33] had lower sequence identity to the UBA*0201 promoter, but both promoters are supported by complete identity to 5'UTR cDNA sequences of bona fida *UBA *alleles suggesting that Atlantic salmon *UBA *alleles have different promoters. The functional consequences of these differences are being investigated.

**Figure 6:**
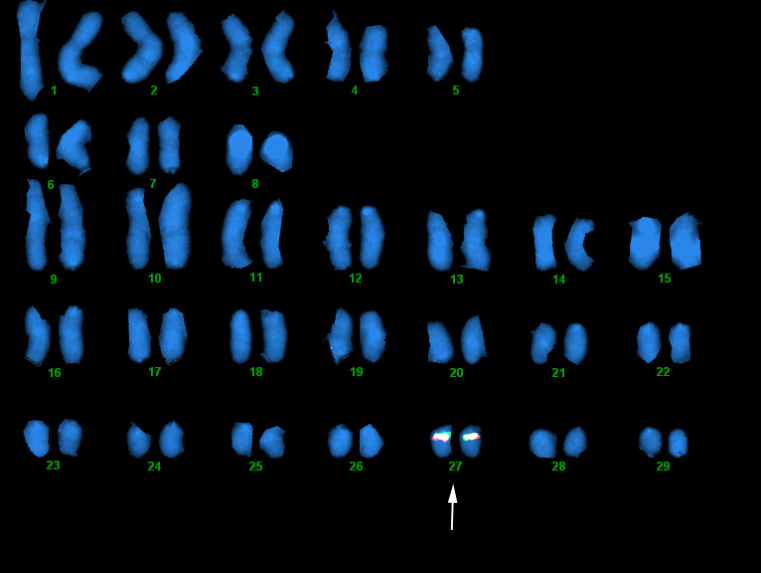
**FISH mapping of BAC clones**. Both 92I04 (in red) and 30C23 (in green) hybridized to one of the smallest chromosome, potentially chromosome 27 defining the Atlantic salmon IA region.

The 30C23/868O01 and 92I04 BACs jointly have an intron sequence of 7 kb while in rainbow trout, the intron between the *UBA *α1 and α2 exons is 18 kb [6] suggesting we lack approximately 11 kb to have a continuous genomic sequence of the entire *UBA *region. PCR and cloning of the gap was performed multiple times, but despite successful PCR amplification no fragments covering the gap have been cloned suggestive of an unclonable region. The amplified products support an intron sequence of approx. 18 kb. Unfortunately no mRNA or cDNA is available from the BAC library fish, preventing verification of expressed *UBA *alleles in this animal. To verify the linkage between 92I04 and 30C23 fluorescent in situ hybridization was undertaken and showed that both BACs hybridized to the same region of one of the smallest chromosomes, potentially chromosome 27 (Fig. 6). The close linkage described by Miller *et al*. [5] between *ULA *and *UBA *also supported 30C23/868O01 being an extension of the IA region.

#### *Sasa-ULA*

The *ULA *locus residing approximately 50 kb upstream of the *UBA *locus matched a partial ULA*0102 sequence [Genbank:DQ091800] described by Miller *et al*. [5]. Another EST in the cGRASP database [34,35] provided us with a full-length match [Genbank:DY699730]. The exon encoding the transmembrane domain is missing, suggestive of a secreted MHC class I molecule (Fig. 4). Similar secretory class I molecules are also found for human class I molecules and the potential role of secretory HLA-G is currently being deciphered and holds promise for an interesting function. The 30C23 *ULA *gene has an α1 exon with highest sequence identity to UBA*0301 while α2 and downstream exons have highest identity to UBA*0801. No ESTs for *ULA *have been identified in rainbow trout, and a negative PCR-based survey for this gene in rainbow trout by Miller *et al*. [5] suggest this gene may be unique to Atlantic salmon.

#### *Sasa-UCAψ*

**Figure 7:**
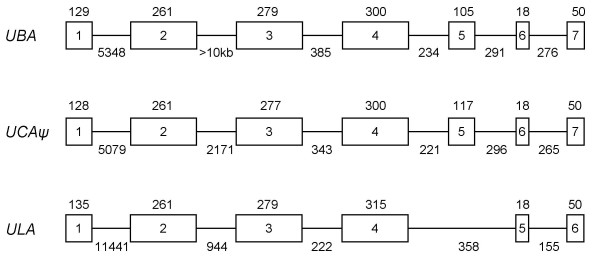
**Exon intron organization of the Atlantic salmon *UBA*, *UCA *ψ and *ULA *loci**. The exons are boxed with the sizes in bp above sequence and intron sizes below.

Only one MHC class I locus was identified in the four BACs representing the IB region. This locus found in 8I14 showed highest sequence identity to the Onmy UCA*0301 allele and was thus denoted *Sasa-UCA*. Multiple salmon ESTs with a polymorphic pattern resembling that of *Onmy-UCA *sequences were found in databases. However, both the 8I14 *UCA *ORF sequence as well as matching ESTs (Table 1), contained an internal stop codon in exon 3 making *Sasa-UCA *an expressed pseudogene. The exon intron organization of the *UBA*, *ULA *and *UCAψ *loci are quite similar apart from the enlarged first intron in *ULA*, the even larger second intron of *UBA *and the missing transmembrane exon of *ULA *(Fig. 7).

### Antigen presenting genes

**Figure 8:**
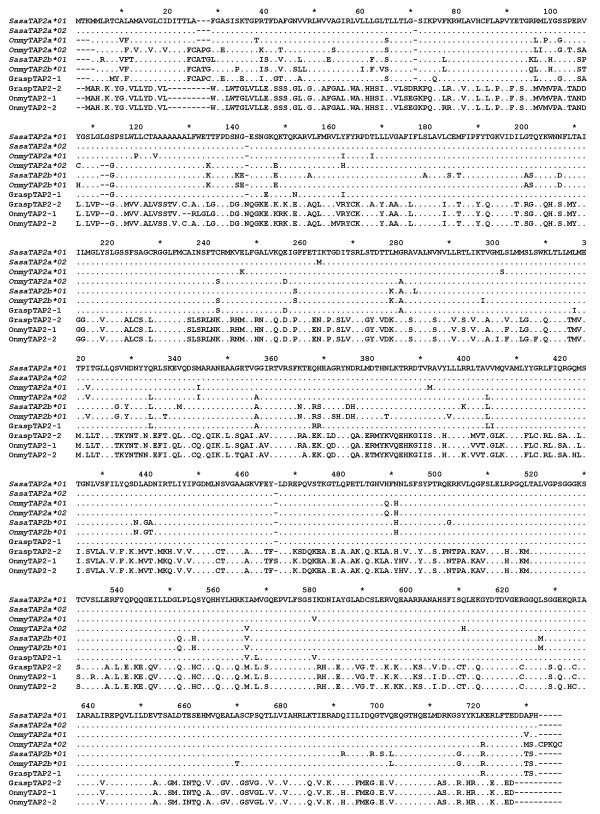
**TAP2 sequence alignment**. A comparison of the amino acid sequences for TAP2s in Atlantic salmon and rainbow trout. SasaTAP2a*01 and SasaTAP2a*02 are from the allelic BACs 714P22 and 92I04 respectively while SasaTAP2b*01 is from the IB region BAC 8I14. GraspTAP2-1 represents an assembly of GRASP EST-clones [Genbank:DW551454, Genbank:DW563627, Genbank:DW563628, Genbank:DW580394 and Genbank:DY730930]), as does GraspTAP2-2 [Genbank:DW544931, Genbank:DW577600, Genbank:DW577601, Genbank:DW580644]. The TAP2 sequences from rainbow trout are OnmyTAP2a*01 [Genbank:AAD53035], OnmyTAP2-1 [Genbank:AAB62237], OnmyTAP2-2 [Genbank:AAD53034], OnmyTAP2a*02 [Genbank:BAD89546] and OnmyTAP2b*01 [Genbank:BAD89558].

Previously reported cDNAs for TAP2, which were assumed to reside in the IA and IB region and denoted *TAP2B *[Genbank:Z83329] and *TAP2A *[Genbank:Z83328] [36] respectively, indeed did match the TAP2 in the IA and IB BAC sequences. To avoid nomenclature confusion we hereby rename our IA *UBA*-linked TAP2 locus to *TAP2a *and the IB U*CAψ *linked TAP2 locus to *TAP2b*. We adapted a similar nomenclature for the reported rainbow trout TAP2 sequences, and suggest that TAP2 sequences without locus identification should be denoted TAP2 only (Fig. 8). Atlantic salmon IA and IB TAP2 sequences have more than 90% aa sequence identity and a similar identity to the rainbow trout IA and IB TAP2 sequences described by Shiina *et al*. [6].

Other TAP2 ESTs were also found in databases, which were difficult to define as *TAP2a *or *TAP2b *variants such as the GraspTAP2-1 in Fig. 8. Attempts to decipher locus origin including rainbow trout information shows that the *TAP2a *and *TAP2b *sequences described by Shiina *et al*. [6] resembles the *TAP2b *sequence identified in Atlantic salmon containing for instance the characteristic FCA motif at position 25 and the two aa deletion at position 110 (Fig. 8). A rainbow trout *TAP2a *(previously denoted TAP2B) [Genbank:AAD53035] sequence described by Hansen *et al*. [37], shown by in situ hybridization to reside in the IA region [8], resembles the Atlantic salmon IA *TAP2a *sequence and does not contain these motifs mentioned above. Thus, rainbow trout has a polymorphic *TAP2a *locus and the confusing sequence identities between the two TAP2 loci may suggest that these genes are exposed to recombination or gene conversion mechanisms. Locus designation of either salmon or trout TAP2 sequences therefore can not be performed on sequence alone, but must be verified by linkage mapping. Other more divergent Atlantic salmon TAP2 ESTs [Genbank:DW580644 and Genbank:DW577601] have approx. 50% sequence identity to all above described IA and IB TAP2 sequences (GraspTAP2-2 in Fig. 8), but has 94% sequence identity to a rainbow trout TAP2 variant described by Hansen *et al*. [37] (previously denoted TAP2A) [Genbank:AF115537]. If these sequences represent an additional TAP2 locus, i.e. a TAP2c locus, or are allelic variants of the TAP2a/b loci is currently unknown. Ancient lineages of divergent MHC class I, TAP1, TAP2 and LMP7 haplotypes have been described in *Xenopus *where the sequence identity between allelic TAP2s was less than 76% [38]. Similar ancient lineages of *UBA *and *TAP2a *may also exist in salmonids, where we were unfortunate enough to sequence allelic variants belonging to similar lineages.

All PSMBs genes in the salmon IA and IB regions were fully intact, as opposed to the IA region in rainbow trout where PSMB8 was a pseudogene, lacking the first three exons. The core region *PSMB8*, *PSMB9, PSMB9-like*, *PSMB10 *and *TAP2 *loci were found to be organized in a similar fashion in both regions and also displayed a high amount of sequence identity (Table 2). Some nucleotide differences were found between the allelic BACs 92I04 and 714P22 genes that resulted in amino acid changes; PSMB10a*01 and PSMB10a*02 had 2 aa differences located in the propeptide at position 12 (T vs. S) and 17 (E vs. K). The proteasome subunits therefore seem to be non-restrictive in providing peptides for *UBA *molecules as opposed to *Xenopus *where PSMB8 (LMP7) segregates in lineages with MHC class I, TAP1 and TAP2 [39].

**Table 2:** Sequence comparison between antigen presenting genes in the MHC IA and IB regions in Atlantic salmon and rainbow trout

GENE	% nt Sasa IA/IB	% aa Sasa IA/IB	% nt IA Sasa/Onmy	% aa IA Sasa/Onmy	% nt IB Sasa/Onmy	% aa IB Sasa/Onmy
*PSMB8*	95	95	-	98	96	98
*PSMB10*	94	92	94	92	97	98
*PSMB9*-like	96	99	97	97	98	99
*PSMB9*	97	100	96	98	98	99
*TAP2*	92	90	94	93	96	95

The table shows comparison of % nucleotide (nt) and % amino acid (aa) identity between the genes found respectively in the Atlantic salmon IA vs IB region and in the IA and IB region of Atlantic salmon versus rainbow trout [6].

Tapasin (TAPBP) is a key member of MHC class I antigen-loading complexes, linking the class I molecule to the TAP. A full-length cDNA [Genbank:DQ451008] recently described by Jorgensen *et al*. [40] matched the *TAPBPa *locus in IA and another EST matched the *TAPBPb *locus in IB. As opposed to Atlantic salmon, the rainbow trout TAPBP in the IB region was described as a pseudogene both by Shiina *et al*. [6] as well as by Landis *et al*. [41] due to a deletion of the last 3 or 4 exons respectively. Landis *et al*. did however find transcripts of the first 4 exons. Different rainbow trout strains were used in the two studies, potentially accounting for the observed differences in deleted exons.

The core IA region in Atlantic salmon, ranging from the UBA α2 exon and downstream including TAP2, shows 87.6% sequence identity over 20289 bp to the same region in rainbow trout. A comparison of the salmon and rainbow trout IB region sequences from PSMB8 to TAP2 show 91.4 % identity over 20331 bp. This would be in accordance with the general perception that UBA lineages are ancient while the polymorphism of the duplicated IB region has evolved after the duplication event.

### Salmonid MHC evolution and function

In the Atlantic salmon IB region we found only one MHC class I pseudo locus denoted *UCAΨ*, which is still being transcribed and shows a polymorphic pattern similar to that of rainbow trout *UCA *and *UDA *[42]. The rainbow trout IB region contained four MHC class I loci denoted *UCA*, *UDA*, *UEA *and *UFAΨ *[6]. As suggested by Shiina *et al*. [6] there has been a primordial salmonid MHC region containing three MHC class I loci (UCA-, UEA – and UBA-like) where UEA and UBA have been deleted from the Atlantic salmon IB region and UCA and UEA have been deleted from the Atlantic salmon IA region. The trout IB *UDA *locus is a duplication of *UCA *that occured in trout only. Once the extended trout IA region is sequenced we will see if the *UBA *to *ULA *duplication occurred in both species and if the UCA and UEA homologues have been retained in this region of trout.

The salmonid whole-genome duplication was estimated to have occurred between 25 and 125 mya [18] while the study of Shiina *et al*. [6] estimate the duplication to have occurred 60 mya based on sequence identity of the MHC class I regions. Evolving from a tetraploid to a diploid state includes not only accumulation of mutations, but also random rearrangements and recombinations as exemplified by the multiple deletions that have occurred in the Atlantic salmon IA and IB regions. With a sequence identity between the Atlantic salmon IA and IB regions of approximately 82 percent, recombination may even be occurring between the two duplicates today. Salmonids are also known for using recombination within the second intron of the *UBA *locus to generate "new" alleles using exons already tested for functionality [2-4]. As recombination was not observed in 800 siblings [43] the recombination frequency is probably low. One way of reducing the risk of recombination between duplicates may be insertions such as the 220 kb insertion with three copies of chitin synthase genes in the IB region.

Another example of differences between Atlantic salmon and rainbow trout is the chromosomal location of the IA region. In both species, the IB region is located on chromosome 14 [6,8] (data not shown for salmon), while the IA region is located on chromosome 18 in rainbow trout and on one of the smaller chromosomes, potentially chromosome 27, in Atlantic salmon (Fig. 6) [8,44]. In Atlantic salmon, the IA and IB regions map to linkage groups 15 and 3 respectively [45], while in rainbow trout they map to linkage groups 16 and 3 [20] supporting the differences. Atlantic salmon and rainbow trout have diploid chromosome numbers ranging from 58 to 64 [46,47]. Most likely, different centric fusions have occurred in the diploidization processes of Atlantic salmon and rainbow trout leading to IA residing on one arm of a metacentric chromosome in rainbow trout while on an acrocentric chromosome in Atlantic salmon.

Why the Atlantic salmon IB region has undergone more deletions than trout is unknown, but it has functional consequences. The IB region has been identified as a major QTL for resistance towards IHNV in Atlantic salmon and IPNV in rainbow trout where the polymorphic *UCA*, *UDA *or *UEA *loci were suggested as prime candidates for the observed effects [23,24]. As our study indicates that the Atlantic salmon IB region only contains a *UCA *pseudolocus, there must either be other genes flanking our BACs which contribute to resistance or there could be haplotype variation in number of class I loci between Norwegian and Canadian Atlantic salmon.

The IA region was not found associated with resistance towards IHNV in Atlantic salmon nor IPNV in rainbow trout. Atlantic salmon *UBA *genotypes have however been shown to provide resistance towards the viral pathogen causing Infectious Salmon Anaemia (ISA) [21,22]. An ongoing study will identify the role of Atlantic salmon IA and IB in providing resistance towards IPNV, enabling us to decipher between differences in pathogens versus genetic organization. Apart from the potential *TAP2a *and *UBA *lineages, limited polymorphism in PSMBs and other linked loci suggest that the observed linkage between *Sasa-UBA *and disease resistance in Norwegian Atlantic salmon [21,22] is caused by *Sasa-UBA *alleles or genotypes and not closely linked genes. However, the PSMBs and TAP2 molecules residing in the IB region might still influence the overall peptide repertoire available for presentation by *UBA *alleles. Due to the pseudo status of *Sasa-UCA*, the PSMBs and TAP2B in the IB region will most likely devolve over time.

## Conclusion

We have characterized the gene organization of more than 500 kb of the two duplicated MHC regions in Atlantic salmon. Although Atlantic salmon and rainbow trout are closely related, the gene organization of their IB region has undergone extensive gene rearrangements. The Atlantic salmon had only one identified MHC class I *UCA *pseudo gene in the IB region while this region in trout contained the four MHC class I loci *UCA*, *UDA*, *UEA *and *UFAψ *. The Atlantic salmon IB region also contained a 220 kb insertion as compared to the IA region potentially limiting recombination between the two regions. The large difference in gene content and most likely function of salmon and trout class IB regions clearly argues that sequencing of salmon will not necessarily provide information relevant for trout and vice versa.

## Methods

### Screening of the BAC library

An Atlantic salmon BAC library (CHORI214) was obtained from CHORI [48]. The library consisted of approximately 300.000 recombinant clones with an average insert size of 190 kb, representing 18-fold genome coverage [49]. All filters in the library were hybridized with two probes containing conserved exons of the *Sasa-UBA *and *Sasa-TAP2 *loci or using over go's as described by Han *et al*. [50]. Both probes were PCR amplified (primers listed in Table 3) from cDNA clones and gel purified, and then radioactively labeled with the Rediprime Random Labelling Kit (Amersham), including spermine precipitation of labeled DNA. Filter hybridizations were performed as described by CHORI. Probed BAC library filters were visualized using a Typhoon Phospho Image Scanner (Amersham).

**Table 3:** Primers used for probes, southern hybridization and PCR

	Sequence (5'-3')	Position	Comments
UBA 540F			
UBA TmR	TCTTCTTCCAAATGACGACCCC	Exon 5	cDNA amplification *Sasa-UBA*
*TAP2*.550F	GCGGGACACCGTCAGGGCAGT	Exon 5	cDNA amplification *Sasa-TAP2a/b*
*TAP2*.850R	CGGCCCCACCAGAGCAGTCAG	Exon 8	cDNA amplification *Sasa-TAP2a/b*
*PSMB8*.50F	GACTTCGTGGGCAGATTCTT	Exon 2	cDNA amplification *Sasa-PSMB8*
*PSMB8*.350R	TCAGCAGCACTACCAGACAT	Exon 3	cDNA amplification *Sasa-PSMB8*
*PSMB9*b4.590F	GAGGACTGCCAACAGTTTGTT	Exon 4	cDNA clone (2B4) amplification *Sasa-PSMB9*
*PSMB9*b4.876R	ATATCATGCTGGCACAATGTT	3' UTR	cDNA clone (2B4) amplification *Sasa-PSMB9*
UBAlead1.F	CTGGGAATAGGCCTTCTACAT	Exon 1	Sasa-UBA*0201,*0801,*0901 specific
UBAlead2.F	AGCCCTACATTCTTCATCTGC	Exon 1	Sasa-UBA*0301,*1001 specific
UBA02.285R	GTTTGATTGAAGCGGGATTTC	Exon 2	Sasa-UBA*0201 specific
UBA03.281R	TCTGATTGAAGCGCTGCTTGG	Exon 2	Sasa-UBA*0301 specific
UBA08.280R	CTGATTGAAGCGCTGCTTGAC	Exon 2	Sasa-UBA*0801 specfic
UBA01.286R	GTTTGATTGAAACGTGTCTG	Exon 2	Sasa-UBA*0901 specific
UBA10.272R	TTGAAAGATTGTGAGGTGCT	Exon 2	Sasa-UBA*1001 specific
ssal.phc-004.039-2	CTCTCCAGTGACCTGCCACGCTACAGGTTTCTATCCCAGTGGAGTCATGGTGTTCTGGCAGAAAGATGGA		MHC Non-classical
ssal.rgb-532.282-r	GCCAATGTGCTCATGGCTATAGTCATCATTGTGTCTGTAGTCTTGATACTCACTGTCCTATTCAAGTATT		MHC Non-classical
ssal.rgb-505.101	GCTACCATCGGCTCAAAGAGGAACTCTTTGAGGACTGATTAGGAATCACACAGCTGTCAGAGAGAGAGAC		TAP2
ssal.rgb-516.329	TCAACCTGTACCACATGCAGGAGGACGGCTGGATAAAGGTGTGTAAGGAGGACGTTTCAGAGCTGATC		PSMB8
ssal.rgb-523.298	GTTGATGGACAGGAAGGGGAGCTACTACAAACTAAGAGAGAGACTGTTCACGGAAGACGACACGTCACAT		TAP2
ssal.rgb-550.202	TTCATTGGCTATGAGTCGAGATGGTTCCAGCGGAGGCGTGGCCTACCTTGTCACTATTGATGAAAAGGGT		PSMB9
868O01_F1	GGCTTGCGGAGTAGAACACTTGAAAAAGAA		GAP-primers
868O01_F2	GCAACCAATACACTGCAGTATTTCTACACGGCCACTTCTGGTATAGATAAC		GAP-primers
868O01_F3	TCAATCAAACTGGAGGTGAGTAGAGACAGA		GAP-primers
714P22_R1	CAGCGGAAACGCAACAAACACAAGAATAACTTACTAACAAATTAGAATCA		GAP-primers
714P22_R2	AGCATACAACCATGCCGACAACCATC		GAP-primers
92I04_R1	AACATTTCAAGGTGGTGAAACTATTCACAT		GAP-primers
92I04_R2	AATTAGTTGTTGTTCACTATGTTAATTAGT		GAP-primers

### Characterization of BACs

MHC class I positive BAC clones were ordered into contigs using restriction fragment analysis together with GRASP *Hind*III fingerprint information [27]. Southern blot analysis of *Not*I (NEB) and *Nru*I (NEB) digested BAC DNA was performed to characterize the clones. The digested DNA was electrophoresed for 16 h and then transferred to Hybond membranes (Amersham). The MHC class I and TAP2 probes described earlier together with probes for PSMB8 and PSMB9 (unpublished data), were used for hybridization to the southern blots. A mixed probe containing 5 *UBA *leader to alpha1 exons amplified from the alleles UBA*0201, *0301, *0801, *0901 and *1001 cDNAs (primers listed in Table 3) was also used. Hybridization with end-labeled Sp6 and T7 oligos were used to orient end-fragments of BAC inserts.

Blots were prehybridized at 65°C for 30 minutes in hybridization buffer (5× SSC, 5× Denhardt's solution and 1% SDS) with. This was followed by replacement with fresh, preheated (65°C) hybridization buffer and the addition of the radio labeled probes. Hybridization was allowed to proceed overnight. Following hybridization, the membranes were washed three times with 20 ml of 2× SSC, 0.1% SDS at 65°C for 30 min. Prehybridization, hybridization and wash conditions were the same for all probes. To further characterize the BACs we used primers spanning a polymorphic (CA)n repeat located in the 3'UTR of the *UBA *locus [3] both on individual BAC DNA as well as on genomic DNA from the animal the library was made from. PCR on genomic DNA from the BAC library animal was performed with GAP-primers (Table 3) with Herculase Enhanced polymerase (Stratagene) according to protocol. Amplified products were ligated into the three different vectors using TOPO-TA Cloning Kit with pCR2.1-TOPO (Invitrogen), TOPO-XL PCR Cloning Kit with pCR-XL-TOPO (Invitrogen) and CloneSmart LCKan Blunt Cloning Kit with pSMART LCKan (Lucigen Corporation) and subsequently transformed into XL-10 Gold cells (Stratagene).

### Sequencing strategy

The selected BACs were subjected to a shotgun sequencing approach. Briefly, BAC DNA was purified by Nucleobond BAC Maxi Kit (BD Biosciences ClonTech). Isolated BAC DNA was nebulized (Invitrogen) (20PSI/15s) and fragments in size range 2–4 kb were purified from agarose gel and blunt-ended with Mung Bean Nuclease, T4 DNA polymerase and Klenow (NEB). Fragments were ligated into a pUC19 vector (Fermentas) cut with *Sma*I, and transformed into XL10-Gold (Stratagene). The sequencing templates were prepared by standard alkaline lysis, and sequencing reactions were run on an ABI3100 or ABI3700 DNA sequencer (Applied Biosystems). Bases were called using Phred [51,52]. High quality sequencing reads were assembled using Phrap, and viewed and edited using Consed [53]. Autofinish [54] was used for closing gaps by designing gap-closing primers with subsequent direct sequencing on BAC DNA or PCR amplification and PCR product sequencing. The BAC sequences were submitted to Genbank and given the following accession numbers: 8I14 (188042 bp, [Genbank:EF427379]), 15L20 (145959 bp [Genbank:EF427378]), 30C23 (218410 bp, [Genbank:EF427381]), 92I04 (128344 bp, [Genbank:EF427384]), 189M18 (170847 bp, [Genbank:EF427377]), 424M17 (163489 bp, [Genbank:EF427382]), 523M19 (188299 bp [Genbank:EF427383]), 714P22 (244579bp, [Genbank:EF210363]), and 868O01 (140046 bp, [Genbank:EF441211]).

### Bioinformatics

DIGIT [55] and GENSCAN [56] were used to predict novel genes and to identify open reading frames. Dotter [57] was used to compare the BAC sequence to itself as well as to other BACs and to identify duplicated regions. Vista was used for sequence comparisons [58]. Blast searches identified possible functions of predicted genes [59]. Sim4 [60] and Spidey [61] were used to adjust exon and intron boundaries aligning EST/cDNA sequences to the BAC sequences. Repeatmasker [62] were used to identify repeats. Multiple sequence alignments of the assumed or verified expressed exons were done using ClustalX [63] followed by manual inspection.

### In situ hybridization and karyotyping

Blood was cultured from the Norwegian strain of Atlantic salmon using standard methods [64]. DNA was isolated from three BAC clones (8I14, 30C23 and 92I04) from the CHORI library using the Qiagen Midi-Preparation kit. These clones were labeled with either Spectrum Orange (Vysis, Inc.) using a nick translation kit (Vysis, Inc.) or digoxigenin according to manufacturers instructions. Human placental DNA (0.2 μg) and Cot-1 DNA (1 μg, prepared from Atlantic salmon) were added to the probe mixture for blocking. Hybridizations were carried out at 37°C overnight and post-hybridization washes were as recommended by the manufacturer (Vysis, Inc.) with minor modifications [65]. Secondary antibodies to Spectrum Orange (Molecular Probes) were used to amplify the signal in some cases. Slides were counter-stained with 4'6'-diamidino-2-phenylindole (DAPI) at a concentration of 125 ng DAPI in 1 ml antifade solution. Images were captured with a Sensys camera and analyzed with Cytovision Genus (Applied Imaging, Inc.) software.

## Authors' contributions

MFL: Performed sequencing, sequence data analysis, annotations and drafted the manuscript.

HH: Performed library screening, sequencing and BAC restriction mapping.

UG contributed to project design, library screening, sequencing and revision of manuscript.

HGB, MBS, GAC and LR: Performed library screening and sequencing.

RBP: Labelled BAC clones and performed fluorescence *in situ *hybridization on salmon chromosomes.

KMM: Identified 30C23 containing ULA, performed some sequence annotation

WSD contributed to the project planning and directions.

BFK contributed to the planning, design, direction and analysis.

All authors read and approved the final manuscript.

## References

[B1] Shiina T, Inoko H, Kulski JK (2004). An update of the HLA genomic region, locus information and disease associations: 2004. Tissue Antigens.

[B2] Aoyagi K, Dijkstra JM, Xia C, Denda I, Ototake M, Hashimoto K, Nakanishi T (2002). Classical MHC class I genes composed of highly divergent sequence lineages share a single locus in rainbow trout (Oncorhynchus mykiss). J Immunol.

[B3] Grimholt U, Drablos F, Jorgensen SM, Hoyheim B, Stet RJM (2002). The Major Histocompatibility Class I locus in Atlantic salmon (Salmo salar L.): Polymorphism, linkage analysis and protein modelling. Immunogenetics.

[B4] Shum BP, Guethlein L, Flodin LR, Adkison MA, Hedrick RP, Nehring RB, Stet RJ, Secombes C, Parham P (2001). Modes of salmonid MHC class I and II evolution differ from the primate paradigm. J Immunol.

[B5] Miller KM, Li S, Ming TJ, Kaukinen KH, Schulze AD (2006). The salmonid MHC class I: more ancient loci uncovered. Immunogenetics.

[B6] Shiina T, Dijkstra JM, Shimizu S, Watanabe A, Yanagiya K, Kiryu I, Fujiwara A, Nishida-Umehara C, Kaba Y, Hirono I, Yoshiura Y, Aoki T, Inoko H, Kulski JK, Ototake M (2005). Interchromosomal duplication of major histocompatibility complex class I regions in rainbow trout (Oncorhynchus mykiss), a species with a presumably recent tetraploid ancestry. Immunogenetics.

[B7] Dijkstra JM, Katagiri T, Hosomichi K, Yanagiya K, Inoko H, Ototake M, Aoki T, Hashimoto K, Shiina T (2007). A third broad lineage of major histocompatibility complex (MHC) class I in teleost fish; MHC class II linkage and processed genes. Immunogenetics.

[B8] Phillips RB, Zimmerman A, Noakes MA, Palti Y, Morasch MR, Eiben L, Ristow SS, Thorgaard GH, Hansen JD (2003). Physical and genetic mapping of the rainbow trout major histocompatibility regions: evidence for duplication of the class I region. Immunogenetics.

[B9] Michalova V, Murray BW, Sultmann H, Klein J (2000). A contig map of the Mhc class I genomic region in the zebrafish reveals ancient synteny.. J Immunol.

[B10] Clark MS, Shaw L, Kelly A, Snell P, Elgar G (2001). Characterization of the MHC class I region of the Japanese pufferfish (Fugu rubripes).. Immunogenetics.

[B11] Matsuo M, Asakawa S, Shimizu N, Kimura H, Nonaka M (2002). Nucleotide sequence of the MHC class I genomic region of a teleost, the medaka (Oryzias latipes).. Immunogenetics.

[B12] Tsukamoto K, Hayashi S, Matsuo M, Nonaka M, Kondo M, Shima MI, Asakawa S, Shimizu N, Nonaka M (2005). Unprecedented intraspecific diversity of the MHC class I region of a teleost medaka, Oryzias latipes. Immunogenetics.

[B13] Bingulac-Popovic J, Figueroa F, Sato A, Talbot WS, Johnson SL, Gates M, Postlethwait JH, Klein J (1997). Mapping of mhc class I and class II regions to different linkage groups in the zebrafish, Danio rerio. Immunogenetics.

[B14] Naruse K, Fukamachi S, Mitani H, Kondo M, Matsuoka T, Kondo S, Hanamura N, Morita Y, Hasegawa K, Nishigaki R, Shimada A, Wada H, Kusakabe T, Suzuki N, Kinoshita M, Kanamori A, Terado T, Kimura H, Nonaka M, Shima A (2000). A detailed linkage map of medaka, Oryzias latipes: comparative genomics and genome evolution. Genetics.

[B15] Sambrook JG, Russel R, Umrania Y, Edwards YJK, Campbell RD, Elgar G, Clark MS (2002). Fugu orthologues of human major histocompatibility complex genes: a genome survey. Immunogenetics.

[B16] Sambrook JG, Figueroa F, Beck S (2005). A genome-wide survey of Major Histocompatibility Complex (MHC) genes and their paralogues in zebrafish. BMC Genomics.

[B17] Kasahara M (1999). Genome dynamics of the major histocompatibility complex: insights from genome paralogy. Immunogenetics.

[B18] Allendorf FW, Thorgaard GH, Turner BJ (1984). Tetraploidy and the evolution of salmonid fishes. Evolutionary Genetics of Fishes.

[B19] Arratia G (1997). Basal teleosts and teleostean phylogeny. Palaeo Ichthyologica.

[B20] Phillips RB, Nichols KM, Dekoning JJ, Morasch MR, Keatley KA, Rexroad C, Gahr SA, Danzmann RG, Drew RE, Thorgaard GH (2006). Assignment of rainbow trout linkage groups to specific chromosomes. Genetics.

[B21] Kjoglum S, Larsen S, Bakke HG, Grimholt U (2006). How specific MHC class I and class II combinations affect disease resistance against infectious salmon anaemia in Atlantic salmon (Salmo salar). Fish Shellfish Immunol.

[B22] Grimholt U, Larsen S, Nordmo R, Midtlyng P, Kjoeglum S, Storset A, Saebo S, Stet RJ (2003). MHC polymorphism and disease resistance in Atlantic salmon (Salmo salar); facing pathogens with single expressed major histocompatibility class I and class II loci. Immunogenetics.

[B23] Miller KM, Winton JR, Schulze AD, Purcell MK, Ming TJ (2004). Major histocompatibility complex loci are associated with susceptibility of Atlantic salmon to infectious hematopoietic necrosis virus. Environ Biol Fishes.

[B24] Ozaki A, Sakamoto T, Khoo S, Nakamura K, Coimbra MR, Akutsu T, Okamoto N (2001). Quantitative trait loci (QTLs) associated with resistance/susceptibility to infectious pancreatic necrosis virus (IPNV) in rainbow trout (Oncorhynchus mykiss). Mol Genet Genomics.

[B25] McKay SJ, Devlin RH, Smith MJ (1996). Phylogeny of Pacific salmon and trout based on growth hormone type-2 and mitocondrial NADH dehydrogenase subunit 3 DNA sequences. Can J Fish Aquatic Sci.

[B26] Kibenge FS, Kibenge MJ, Groman D, McGeachy S (2006). In vivo correlates of infectious salmon anemia virus pathogenesis in fish. J Gen Virol.

[B27] Ng SH, Artieri CG, Bosdet IE, Chiu R, Danzmann RG, Davidson WS, Ferguson MM, Fjell CD, Hoyheim B, Jones SJ, de Jong PJ, Koop BF, Krzywinski MI, Lubieniecki K, Marra MA, Mitchell LA, Mathewson C, Osoegawa K, Parisotto SE, Phillips RB, Rise ML, von Schalburg KR, Schein JE, Shin H, Siddiqui A, Thorsen J, Wye N, Yang G, Zhu B (2005). A physical map of the genome of Atlantic salmon, Salmo salar. Genomics.

[B28] (2007). HUGO Gene Nomenclature Committee. Internet.

[B29] Dijkstra JM, Yoshiura Y, Kiryu I, Aoyagi K, Kollner B, Fischer U, Nakanishi T, Ototake M (2003). The promoter of the classical MHC class I locus in rainbow trout (Oncorhynchus mykiss). Fish & Shellfish Immunology.

[B30] Gobin SJ, Peijnenburg A, Keijsers V, van den Elsen PJ (1997). Site alpha is crucial for two routes of IFN gamma-induced MHC class I transactivation: the ISRE-mediated route and a novel pathway involving CIITA. Immunity.

[B31] van den Elsen PJ, Peijnenburg A, Van Eggermond MC, Gobin SJ (1998). Shared regulatory elements in the promoters of MHC class I and class II genes. Immunol Today.

[B32] Patikoglou GA, Kim JL, Sun L, Yang SH, Kodadek T, Burley SK (1999). TATA element recognition by the TATA box-binding protein has been conserved throughout evolution. Genes Dev.

[B33] Jorgensen SM, Lyng-Syvertsen B, Lukacs M, Grimholt U, Gjoen T (2006). Expression of MHC class I pathway genes in response to infectious salmon anaemia virus in Atlantic salmon (Salmo salar L.) cells. Fish Shellfish Immunol.

[B34] (2007). Consortium for Genomics Research on All Salmon. Internet.

[B35] Rise ML, von Schalburg KR, Brown GD, Mawer MA, Devlin RH, Kuipers N, Busby M, Beetz-Sargent M, Alberto R, Gibbs AR, Hunt P, Shukin R, Zeznik JA, Nelson C, Jones SR, Smailus DE, Jones SJ, Schein JE, Marra MA, Butterfield YS, Stott JM, Ng SH, Davidson WS, Koop BF (2004). Development and application of a salmonid EST database and cDNA microarray: data mining and interspecific hybridization characteristics. Genome Res.

[B36] Grimholt U (1997). Transport-associated proteins in Atlantic salmon (Salmo salar). Immunogenetics.

[B37] Hansen JD, Strassburger P, Thorgaard GH, Young WP, Du PL (1999). Expression, linkage, and polymorphism of MHC-related genes in rainbow trout, Oncorhynchus mykiss. J Immunol.

[B38] Ohta Y, Powis SJ, Lohr RL, Nonaka M, Pasquier LD, Flajnik MF (2003). Two highly divergent ancient allelic lineages of the transporter associated with antigen processing (TAP) gene in Xenopus: further evidence for co-evolution among MHC class I region genes. Eur J Immunol.

[B39] Ohta Y, Haliniewski DE, Hansen J, Flajnik MF (1999). Isolation of transporter associated with antigen processing genes, TAP1 and TAP2, from the horned shark Heterodontus francisci. Immunogenetics.

[B40] Jorgensen SM, Grimholt U, Gjoen T (2007). Cloning and expression analysis of an Atlantic salmon (Salmo salar L.) tapasin gene. Dev Comp Immunol.

[B41] Landis ED, Palti Y, Dekoning J, Drew R, Phillips RB, Hansen JD (2006). Identification and regulatory analysis of rainbow trout tapasin and tapasin-related genes.. Immunogenetics.

[B42] Dijkstra JM, Kiryu I, Yoshiura Y, Kumanovics A, Kohara M, Hayashi N, Ototake M (2006). Polymorphism of two very similar MHC class Ib loci in rainbow trout (Oncorhynchus mykiss). Immunogenetics.

[B43] Kiryu I, Dijkstra JM, Sarder RI, Fujiwara A, Yoshiura Y, Ototake M (2005). New MHC class Ia domain lineages in rainbow trout (Oncorhynchus mykiss) which are shared with other fish species.. Fish & Shellfish Immunology.

[B44] Fujiwara A, Kiryu I, Dijkstra JM, Yoshiura Y, Nishida-Umehara C, Ototake M (2003). Chromosome mapping of MHC class I in rainbow trout (Oncorhynchus mykiss). Fish & Shellfish Immunology.

[B45] (2007). Salmon Genome Project. Internet.

[B46] Phillips R, Rab P (2001). Chromosome evolution in the Salmonidae (Pisces): an update. Biol Rev Camb Philos Soc.

[B47] Hartley SE (1987). The chromosomes of salmonid fishes. Biol Rev Camb Philos Soc.

[B48] (2007). Children's Hospital Oakland Research Institute (CHORI). Internet.

[B49] Thorsen J, Zhu B, Frengen E, Osoegawa K, de Jong PJ, Koop BF, Davidson WS, Hoyheim B (2005). A highly redundant BAC library of Atlantic salmon (Salmo salar): an important tool for salmon projects. BMC Genomics.

[B50] Han CS, Sutherland RD, Jewett PB, Campbell ML, Meincke LJ, Tesmer JG, Mundt MO, Fawcett JJ, Kim UJ, Deaven LL, Doggett NA (2000). Construction of a BAC contig map of chromosome 16q by two-dimensional overgo hybridization. Genome Res.

[B51] Ewing B, Hillier L, Wendl MC, Green P (1998). Base-calling of automated sequencer traces using phred. I. Accuracy assessment. Genome Res.

[B52] Ewing B, Green P (1998). Base-calling of automated sequencer traces using phred. II. Error probabilities. Genome Res.

[B53] Gordon D, Abajian C, Green P (1998). Consed: a graphical tool for sequence finishing. Genome Res.

[B54] Gordon D, Desmarais C, Green P (2001). Automated finishing with autofinish. Genome Res.

[B55] (2007). Digit Web Server. Internet.

[B56] Burge C, Karlin S (1997). Prediction of complete gene structures in human genomic DNA. J Mol Biol.

[B57] Sonnhammer EL, Durbin R (1995). A dot-matrix program with dynamic threshold control suited for genomic DNA and protein sequence analysis. Gene.

[B58] Frazer KA, Pachter L, Poliakov A, Rubin EM, Dubchak I (2004). VISTA: computational tools for comparative genomics. Nucleic Acids Res.

[B59] Altschul SF, Gish W, Miller W, Myers EW, Lipman DJ (1990). Basic local alignment search tool. J Mol Biol.

[B60] Florea L, Hartzell G, Zhang Z, Rubin GM, Miller W (1998). A computer program for aligning a cDNA sequence with a genomic DNA sequence. Genome Res.

[B61] Wheelan SJ, Church DM, Ostell JM (2001). Spidey: a tool for mRNA-to-genomic alignments. Genome Res.

[B62] (2007). Repeatmasker. Internet.

[B63] Thomson JD, Higgins DG, Gibson TJ (1994). ClustalW: improving the sensitivity of progressive multiple sequence alignments through sequence weighting, position-specific gap penalties and weight matrix choice. Nucleic Acids Res.

[B64] Reed KM, Phillips RB (1995). Molecular cytogenetic analysis of the double-CMA3 chromosome of lake trout, Salvelinus namaycush. Cytogenet Cell Genet.

[B65] Phillips RB, Reed KM (2000). Localization of repetitive DNAs to zebrafish (Danio rerio) chromosomes by fluorescence in situ hybridization (FISH). Chromosome Res.

